# A New Species of *Radula* (Radulaceae, Porellales) From Mid‐Cretaceous Kachin Amber

**DOI:** 10.1002/ece3.72007

**Published:** 2025-08-14

**Authors:** Xiangbo Song, Wen Ye, Zixi Wang

**Affiliations:** ^1^ State Key Laboratory of Palaeobiology and Stratigraphy, Nanjing Institute of Geology and Palaeontology Chinese Academy of Sciences Nanjing China; ^2^ University of Chinese Academy of Sciences Beijing China; ^3^ State Key Laboratory of Cellular Stress Biology, School of Life Sciences Xiamen University Xiamen China

**Keywords:** cretaceous, fossil, liverworts, Myanmar

## Abstract

The liverwort genus *Radula*, one of the largest genera within the order Porellales, is phylogenetically isolated and widely distributed, with many species thriving as epiphytes in diverse ecosystems. Fossil records of *Radula* are scarce, particularly those with sufficient morphological details for precise taxonomic assignment. In this study, we describe and illustrate a new species, *Radula kachinensis* sp. nov., from mid‐Cretaceous Kachin amber in Myanmar. The fossil exhibits a unique combination of diagnostic features, including broad lobules, short keels, and amentulose axes, with amentulose shoots producing 1–2 additional amentulose shoots. The new discovery sheds light on species diversity during the Cretaceous and offers deeper insights into the morphological traits of this liverwort genus.

## Graphical abstract

Fossil records of Radula are scarce, particularly those with sufficient morphological details for precise taxonomic assignment. In this study, we describe and illustrate a new species, *Radula kachinensis* sp. nov., from mid‐Cretaceous Kachin amber in Myanmar. The fossil material exhibits exceptional three orders of branching, an especially uncommon occurrence within Radulaceae.

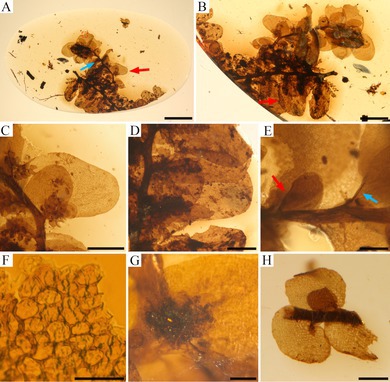

## 1 Introduction

Liverworts thrive in various ecosystems, especially in humid environments, growing on rocks or as epiphytes on bark and leaves (Renner et al. 2010; Gradstein et al. 2001; Goffinet and Shaw 2009). Radulaceae Müller represents one of the most isolated lineages of leafy liverworts (Gradstein et al. 2001; Crandall‐Stotler et al. 2009; Oliveira‐da‐Silva et al. 2021). The family is characterized by unique morphological features, including *Radula*‐type branches, bilobed leaves with a ventral lobule slightly inflated near the keel, the absence of underleaves, and rhizoid bundles on the lobule surface rather than the stem and dorsiventrally flattened perianths (Schuster 1980; Renner 2005; Bechteler et al. 2017; Heinrichs et al. 2018; Renner et al. 2022). Based on both molecular and morphological evidence, the family Radulaceae is currently classified into three genera: *Radula* Dumortier, *Cladoradula* (Spruce) M.A.M. Renner et al., and *Dactyloradula* (Devos et al.) M.A.M. Renner and Gradstein (Renner et al. 2022). *Radula* is the largest genus within the family, comprising between 200 and 350 extant species (Söderström et al. 2016; Patiño et al. 2017; Renner et al. 2022). *Radula* is primarily distinguished from the other two genera by its longitudinal lobule insertion and the absence of a subepidermal layer in the stem, in contrast to the transverse or oblique insertion and the presence of a subepidermis in *Cladoradula* and *Dactyloradula* (Renner et al. 2022). More recently, spore microanatomy has been considered in relation to the classification of Radulaceae. However, the groups identified based on spore characteristics do not fully align with the generic and infrageneric circumscriptions supported by molecular and phylogenetic evidence (Oliveira‐da‐Silva et al. 2024). *Radula* exhibits a wide distribution, from the Arctic to the Antarctic, with its highest diversity in tropical regions (Devos, Renner, Gradstein, Shaw, Laenen, and Vanderpoorten 2011). This genus often co‐occurs with other epiphytic liverworts and diverse moss species. Fossil records from mid‐Cretaceous Myanmar amber show that *Radula* is preserved alongside members of Frullaniaceae Lorch (Figure 3A,H) and Racopilaceae Kindberg in the same amber pieces, suggesting that these bryophytes likely shared the same microhabitat and possibly lived on the same tree (Stilwell et al. 2020; Feldberg, Schaefer‐Verwimp, et al. 2021; Wang et al. 2022).

**FIGURE 1:**
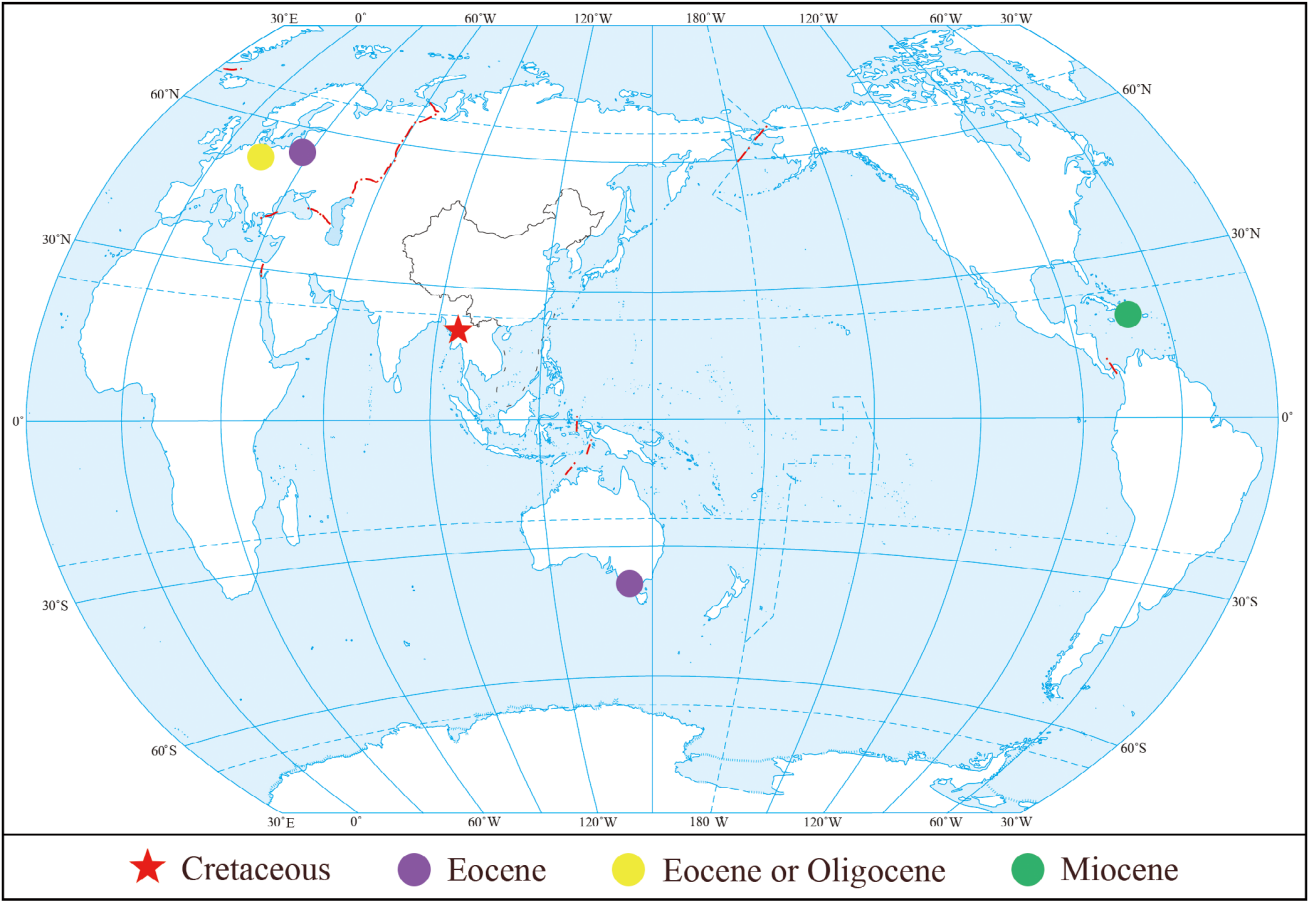
Worldwide distribution of fossil *Radula* from amber deposits. The red star indicates mid‐Cretaceous Mynamar amber, purple circles indicate Eocene Baltic amber and amber deposits from Australia, the yellow circle indicates Eocene or Oligocene Bitterfeld amber, and the green circle indicates Miocene Dominican amber. Base map from the Ministry of Natural Resources, China.

Research on *Radula* has predominantly focused on extant species, with fossil records being relatively rare and fragmented. Most fossil records have been reported within the past decade, primarily from Cretaceous and Cenozoic amber deposits. Most liverwort fossils are preserved as small fragments and often lack the morphological characteristics required for precise taxonomic identification (Heinrichs et al. 2018; Feldberg, Gradstein, et al. 2021; Feldberg, Schaefer‐Verwimp, et al. 2021). To date, 11 fossil species of *Radula* have been reported, including four extinct species from Cretaceous Burmese amber (Bechteler et al. 2017; Feldberg, Schaefer‐Verwimp, et al. 2021; Feldberg et al. 2022) and five extinct species from Cenozoic Baltic and Dominican ambers (Figure 1; Grolle 1987; Heinrichs et al. 2016; Kaasalainen et al. 2016). In addition, two undefined species from the late middle Eocene Alcoa Mine in Victoria, Australia, were tentatively assigned to *Radula* (Stilwell et al. 2020). The richest source of Cretaceous leafy liverworts is the mid‐Cretaceous Kachin amber of Myanmar (Grimaldi et al. 2002; Shi et al. 2012; Yu et al. 2019). Meanwhile, the fossil records of *Radula* are more abundant in the Cretaceous than in other geological periods. Currently, ten fossil species of Porellales have been described from Kachin amber, assigned to Frullaniaceae, Lepidolaeanaceae, and Radulaceae, respectively (Ross 2024). Their well‐preserved details have enabled their assignment to extant families and genera (Feldberg, Schaefer‐Verwimp, et al. 2021). However, the taxonomy of *Radula* remains problematic due to its complex patterns of variation and morphological homoplasy (Wang et al. 2022). For instance, quantitative morphological analyses have shown that lobule shapes are similar across distantly related *Radula* lineages (Renner 2015). Thus, by comparing the fossil features with those of extant species, such as the similarity between *R. cretacea* and the extant representatives of subgenus *Odontoradula* sect. *Acutifoliae* Castle and Grolle ser. *Acutifoliae* Yamada, it is possible not only to evaluate the fossil's classification by comparing divergence time estimates obtained by calibrating different phylogenetic nodes, but also to provide evidence for a late Cretaceous origin of most subgenera and a Paleogene diversification of crown groups (Bechteler et al. 2017; Feldberg, Gradstein, et al. 2021).

In this study, we provide morphological descriptions and detailed illustrations of a new fossil species of *Radula* from mid‐Cretaceous Kachin amber and discuss the possible relationships of the fossil to extant subgenera. This discovery increases our understanding of the morphological characteristics of *Radula* and highlights the species diversity of liverworts during the Cretaceous period.

## 2 Material and Methods

The Kachin amber specimen studied here originates from the Hukawng Valley, Kachin State of northern Myanmar. The age of Kachin amber is estimated to be from the earliest Cenomanian (98.79 ± 0.62 Ma), inferred from radiometric zircon dating and palaeontological evidence (Shi et al. 2012; Mao et al. 2018; Yu et al. 2019).

The amber piece was manually ground and polished using a series of emery papers of different grit sizes, and finally with rare earth polishing powder to produce smooth surfaces for investigation. Amber inclusions were examined and photographed under a Zeiss Discovery V16 stereomicroscope equipped with an Axiocam 512 color digital camera, and a Zeiss Axio Imager 2 light microscope combined with a fluorescence imaging system. Images were stacked with Helicon Focus 7.0.2 and Adobe Photoshop 2021, and further processed in Adobe Photoshop 2021 to adjust brightness and contrast. The studied fossil specimen is housed in the Nanjing Institute of Geology and Paleontology, Chinese Academy of Sciences, Nanjing, China (PB205750). Morphological terminology follows Gradstein et al. (2001) and Renner (2005).

## 3 Systematic Paleontology

**Class Jungermanniopsida Stotler and Crandall‐Stotler, 1977**.

**Order Porellales Schljakov, 1972**.

**Family Radulaceae Müller, 1909**.

**Genus *Radula* Dumortier, 1822**.

**Subgenus *Amentuloradula* Devos et al., 2011**.

**FIGURE 2:**
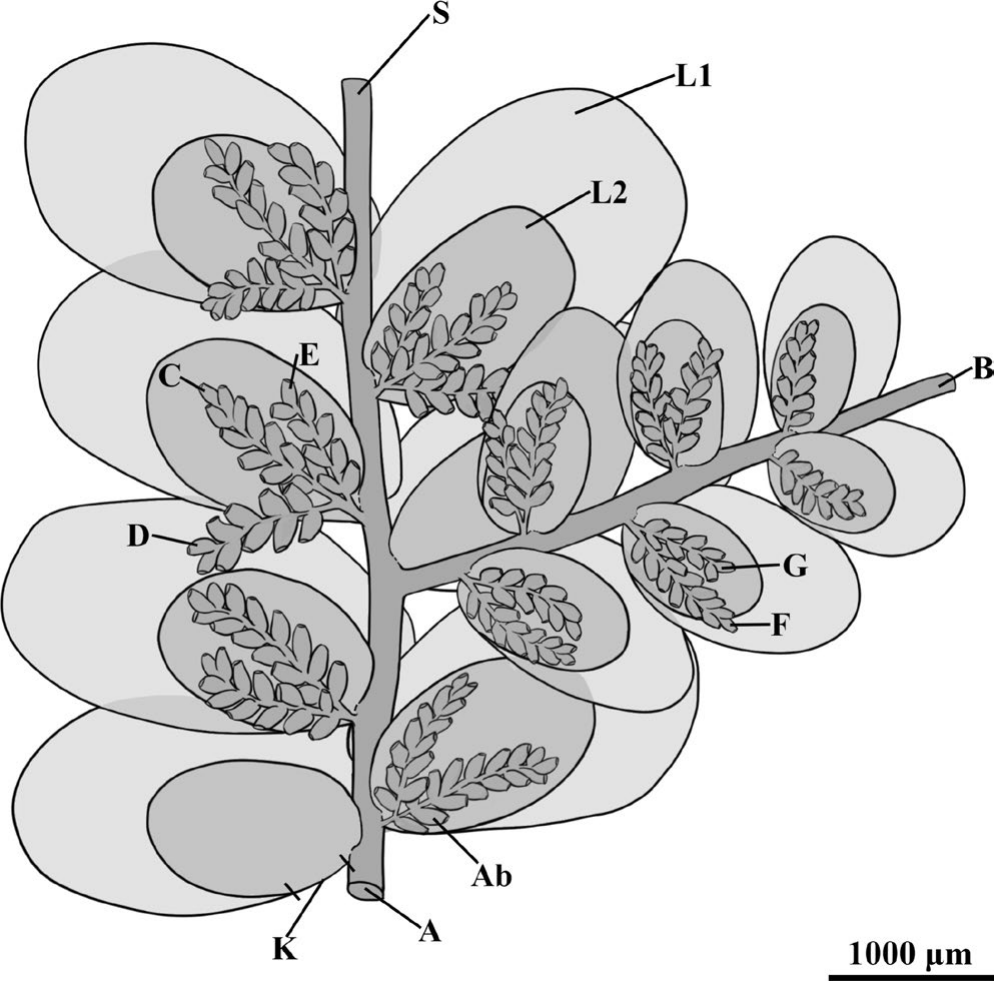
Reconstruction of sterile branches of *Radula kachinensis* sp. nov., in ventral view. Ab: Amentulose branches; L1: Leaf‐lobe; L2: Leaf‐lobule; K: Keel; S: Stem; (A) Primary shoot; (B) Lateral non‐amentulose shoot arises from primary shoot; (C) Amentulose axes arise from primary shoot; (D, E) Additional amentulose branches arise from the amentulose axes; (F) Amentulose branches arise from lateral non‐amentulose shoot. (G) Additional amentulose branches arise from amentulose axes.

**FIGURE 3:**
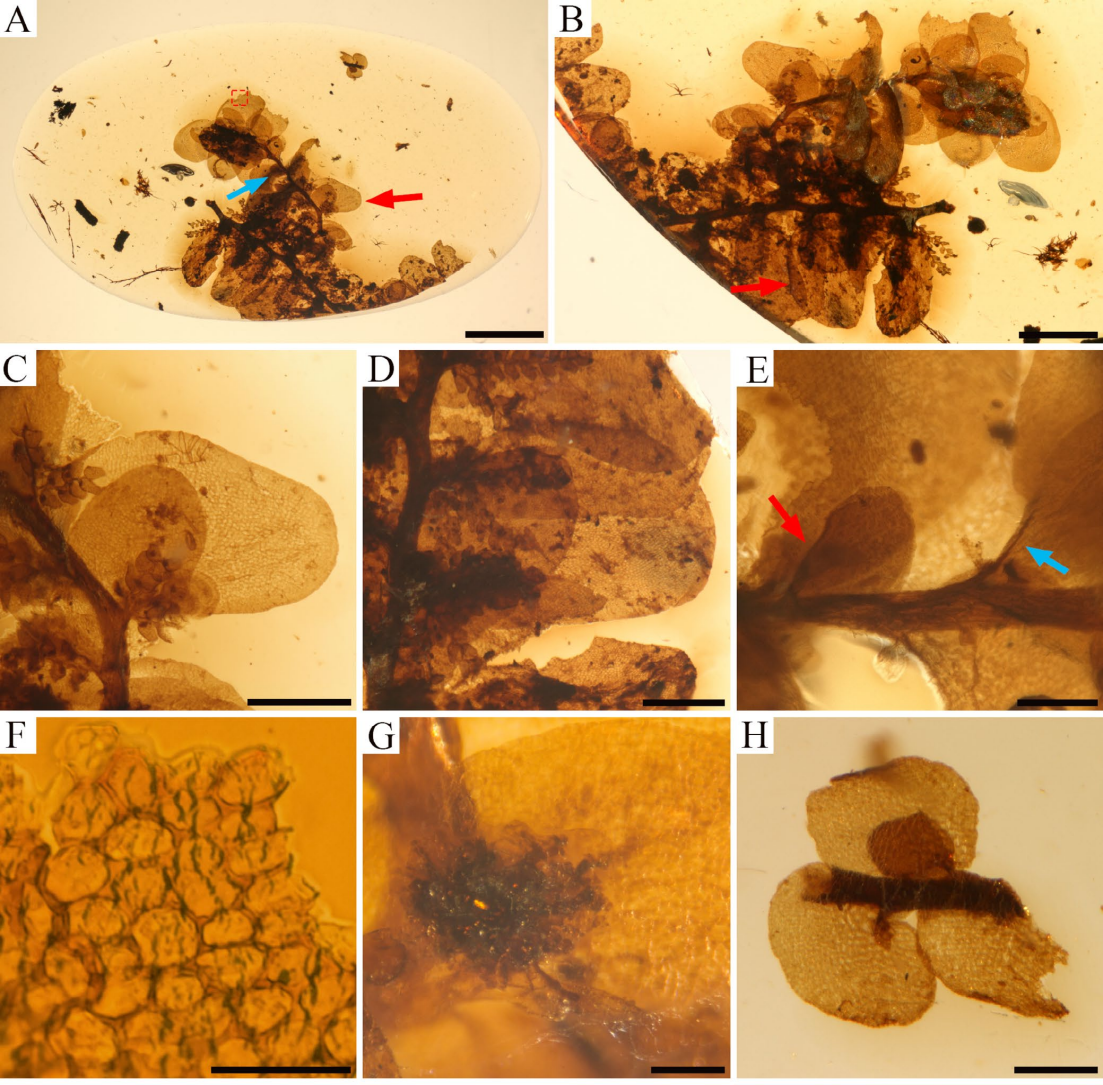
Holotype of *Radula kachinensis* sp. nov. (PB205750). (A) Over view of the fossil. (B) Fragment of gametophyte in dorsal view. (C) Lobe and lobule on secondary branches (marked with red arrowhead in A). (D) Lobe and lobule on primary shoot (marked with red arrowhead in B). (E) The red arrow indicates partially developed leaves, the blue arrow indicates the keel. (F) Cell wall architecture appears conspicuously thickened with angular trigones (enlargement form A). (G) Rhizoids fused together to form a compact structure, originate from the ventral side of lobules on the secondary branches. (H) A fragment of Frullaniaceae is included with *Radula kachinensis* sp. *nov*. Scale bars: 2000 μm in (A); 1000 μm in (B); 500 μm in (C, D); 200 μm in (E, H); 100 μm in (G); 50 μm in (F).

***Radula kachinensis* X.B. Song, W. Ye, and Z.‐X. Wang, sp. nov**. (Figures 2 and 3).

**Holotype**. PB205750, housed in Nanjing Institute of Geology and Paleontology, Chinese Academy of Sciences, Nanjing, China.

**Etymology**. The specific epithet is derived from the Kachin Province in northern Myanmar, where the amber containing the new liverwort was collected.

**Diagnosis**. Sterile shoots with amentulose shoot system exhibiting three orders of branching. Amentulose axes branched, wherein each amentulose shoot has 1–2 additional amentulose shoots. Dorsal lobes large with rounded apices and ventral lobules broadly ovate. Lobule insertion longitudinal on the stem; lobule 1/2–2/3 as wide and ca. 1/2 as long as the lobe. Cell wall architecture conspicuously thickened with angular trigones. Keel short, less than 1/4 the length of thelobe.

**Locality and horizon**. Amber mine located near Noije Bum Village, Tanai Township, Myitkyina District, Kachin State, Myanmar; unnamed horizon, mid‐Cretaceous, ~99 Ma.

**FIGURE 4:**
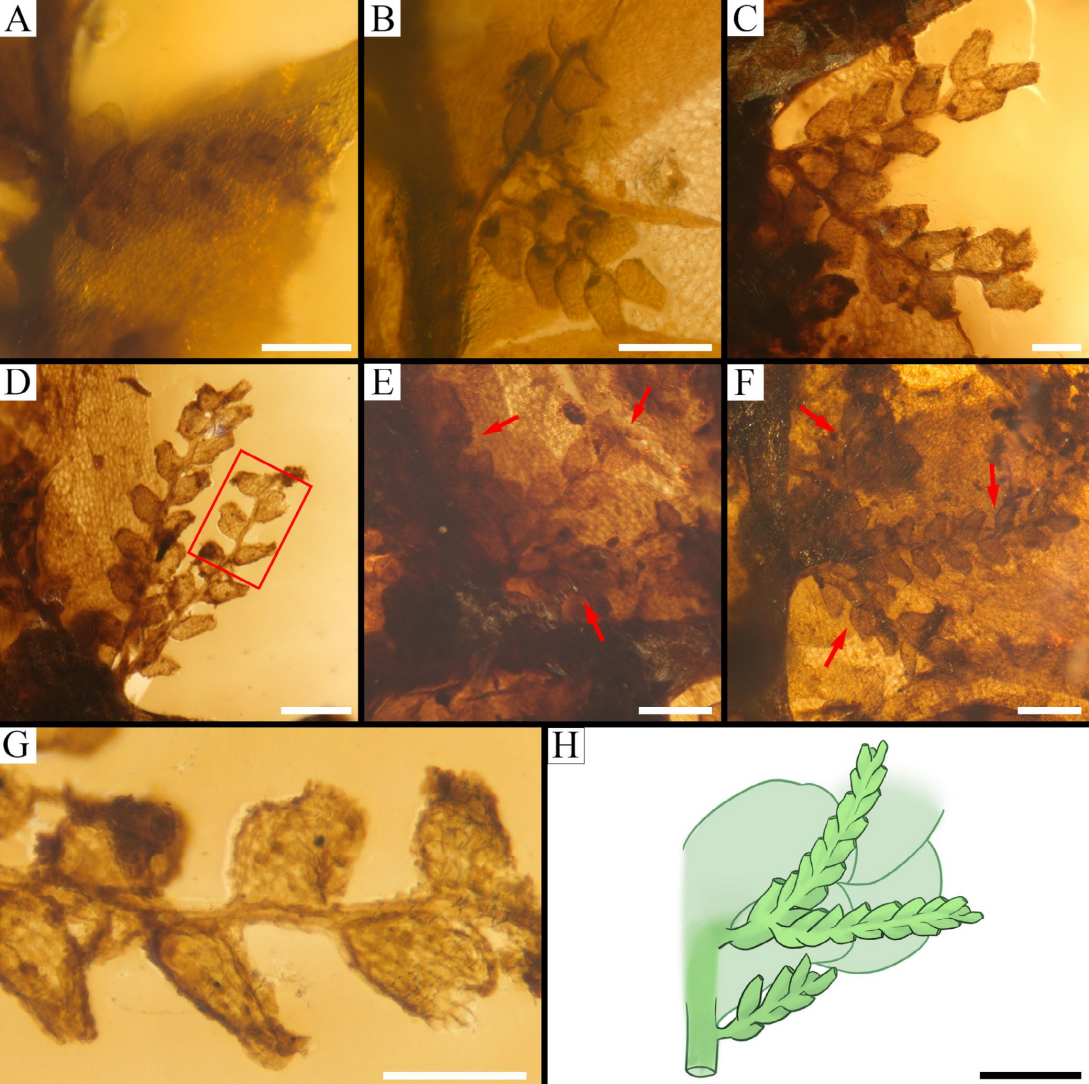
Amentulose branches of *Radula kachinensis* sp. nov. and related extant representatives in *Radula*. (A) An amentulose branch on the secondary shoot. (B) Amentulose shoots bear one lateral branches on the secondary shoot. (C, D) Amentulose shoots bear one lateral branches on the primary shoot. (E, F) The red arrows indicate that amentulose shoots bear two lateral branches on the primary shoot. (G) Details of lobe cells on amentulose branches (enlargement from D). (H) Reconstructions of amentulose branches of *Radula amentulosa* (revised from (Promma et al. 2025)). Scale bars: 500 μm in (H); 200 μm in (A, D, E, F); 100 μm in (B, C, G).

**Description**. Gametophyte fragment sterile, creeping, brown to yellowish brown, 6.55 mm long, 4.88 mm wide with leaves. Primary shoot 4.8 mm long, 3.0–3.9 mm wide with leaves. Secondary vegetative shoots are all lateral branches arising from the primary shoot and include both amentulose axes and *Radula*‐type non‐amentulose shoot, which measure 5.4 mm long, 2.6–2.7 mm wide with leaves (Figure 3A,B). Tertiary branches consist of additional amentulose shoots arising from these secondary amentulose axes. On the primary shoot, each amentulose axis bears one or two further amentulose branches, whereas on secondary shoots, amentulose axes are either unbranched or bear only one additional branch (Figure 4A–F). Amentulose branches do not occur at every stem leaf base and are irregularly arranged. Stem brown to dark brown, slightly zig‐zagged, 4.89 mm long, 0.1–0.19 mm in diameter on the primary shoot; 4.8 mm long, 93–212 μm in diameter on the secondary shoot (Figure 3D,E); surface cells of the stem indistinguishable. Foliation incubous, slightly oblique with lateral leaves alternate (Figure 3A,B,D); leaves longitudinal along the lateral stem surface, dorsal leaf free strip unclear. Dorsal leaf lobes imbricate, broadly elliptic to ovate, slightly oblique in their spread, entire‐margined with predominantly rounded apex; lobes cover and extend onto the dorsal stem surface up to 1–2 times the stem width (Figure 3C,D); dorsal base of the leaf lobe not auriculate and without appendage; antical and interior margin regularly arched, with the postical margin nearly perpendicular to the stem; leaf lobe dimensions 1.8–2.0 mm long, 1.0–1.3 mm wide on the primary shoot, 1.0–1.7 mm long, 0.6–1.0 mm wide on the secondary branch. Marginal leaf lobe cells quadrate to rectangular, 10–20 mm long, 15–25 mm wide, long axis parallel with the leaf margin, free exterior walls of marginal cells unthickened; median portion cells wall isodiametric to slightly elongate or hexagonal with a smooth surface, measuring 16–24 μm, 10–15 μm wide, irregularly sized and arranged; basal cells similar to mid‐lobe cells; the cell wall architecture appears prominently thickened with angular trigones (Figure 3F). Ventral leaf lobules elliptic‐oblong with rounded apex, obliquely spreading, ca. 1/2 of lobe length, 1.2 mm long and 1.0 mm wide on the primary shoot; 0.7–0.9 mm long, 0.45–0.62 mm wide on the secondary branch; antical and postical margin regularly arched, inner margin covers the stem for ca. 1/2 of the stem width (Figure 3C,D). Keels on the primary shoot unclear, but short and straight on the secondary shoot and ca. 1/4 times as long as the lobe (Figure 3E). Amentulose shoots bear one or two lateral branches originating from the base (Figure 4B–F), usually arising from one side of the parent shoot but sometimes from both sides but do not branch near the apex of the parent shoot (Figure 4A); each branch bears approximately 3–7 pairs (or more) of typical fusiform reduced leaves, 168–220 μm wide with leaves. Stem on amentulose shoots slightly zig‐zagged, apices of the stem obviously zig‐zagged; 0.9 mm long, 13–24 μm in diameter angle between two amentulose branches 30°–90° (Figure 4G). Amentulose leaves alternate, isolobous, with margins recurved (Figures 3C and 4A–F). Cells of the leaf margin rectangular, 7.7 μm in diameter; cells of its median portion elongated, 10.8 μm in length. Rhizoids exhibited with compact structure and not occurring at the base of every stem leaf (Figure 3G). Oil bodies are not observed. Asexual reproduction is lacking.

## 4 Discussion

The new material exhibits amentulose branches, which are an important diagnostic feature of the Radulaceae. Amentulose branches are found in extant species of *Dactyloradula*, *Radula* subg. *Amentuloradula* Devos et al., and the only known fossil species, *Radula heinrichsii* K. Feldberg et al. (Devos, Renner, Gradstein, Shaw, and Vanderpoorten 2011; Feldberg, Schaefer‐Verwimp, et al. 2021; Renner et al. 2022; Wang et al. 2022). *Dactyloradula brunnea* (Stephani) M.A.M. Renner and Gradstein, the sole species in the genus *Dactyloradula*, also has amentulose branches, but can be easily distinguished by the presence of blunt teeth or finger‐like appendages at the base of the interior margin (Yamada 1979; Renner et al. 2022). Over 20 extant species of subg. *Amentuloradula* possess amentulose branches, while only a few species, such as *R. amentulosa* Mitten, *R. hicksiae* K. Yamada, *R. splendida* M.A.M. Renner and Devos, *R*. *yangioides* Promma et al., and *R*. *resupinatiramosa* Promma et al., exhibit amentulose shoots that branch. However, except for *R. amentulosa* and *R*. *resupinatiramosa*, all these species feature rectangular or subrectangular lobules (Devos, Renner, Gradstein, Shaw, and Vanderpoorten 2011; Promma et al. 2025). *Radula amentulosa* is identified by the presence of amentulose branches at the base of every stem‐leaf, which are usually unbranched, or if they branch, the branching is irregular (Figure 4H). The lobules are obovate‐rectangular or ovate‐rectangular with a sinuate or nearly straight keel, approximately 1/2–3/5 of the lobe width (Promma et al. 2025). In *R*. *resupinatiramosa*, the amentulose shoots are regularly branched, each producing 1 or 2 additional amentulose shoots by *Radula*‐type branching at the base of the first; one of these additional amentulose shoots is dorsally resupinate (Promma et al. 2025). The new fossil exhibits branched amentulose branches that typically arise from one side of the parent shoot, though occasionally from both sides. Moreover, the new fossil is characterized by large ovate lobules relative to the lobes and short keels.

Currently, only 11 fossil species of *Radula* have been identified worldwide, and most of them can be easily distinguished from the new fossil specimen (Table 1). *Radula cretacea* Bechteler et al. and *R. sphaerocarpoides* Grolle are distinguished from the new fossil specimen by their sharply acute leaf apex and distinctly rounded leaf shapes, respectively (Heinrichs et al. 2016; Bechteler et al. 2017). *Radula baltica* Heinrichs et al., *R. oblongifolia* Caspary et al., and *R. steerei* Grolle are characterized by elliptic lobes and quadrate or rectangular lobules, differentiating them from the new fossil specimen (Grolle 1987; Heinrichs et al. 2016; Kaasalainen et al. 2016). *Radula patrickmuelleri* K. Feldberg et al., displays exceptionally small lobules that reach merely 1/5 the length of its lobes, contrasting with the much larger lobules of the new fossil (Feldberg et al. 2022). *Radula tanaiensis* K. Feldberg et al. features long keels with deeply emarginated lobes at the ends of the keels and lacks amentulose branches (Feldberg et al. 2022), while our fossil shows relatively short keels. *Radula heinrichsii* has amentulose branches, broadly rounded leaf, and slightly zig‐zagged stem, bearing a strong morphological resemblance to the new fossil (Feldberg, Schaefer‐Verwimp, et al. 2021; Wang et al. 2022). However, the amentulose branches in *R*. *heinrichsii* differ from those in the new fossil in that *R*. *heinrichsii* does not branch but is regularly bipinnate at the base of each lateral leaf on the primary shoot. They also differ in the shape of the keel, cell wall structure, and lobule shape. The new fossil specimen has relatively short and straight keels, conspicuously thickened lobe cell walls with angular trigones, and larger oblong lobules, whereas *R*. *heinrichsii* exhibits slightly thickened cell walls and rounded to obovate lobules. Its keels are distinctly emarginated at the ends, approximately 1/3–1/4 the length of the leaf lobe. In conclusion, as the new fossil exhibits significant morphological divergence from the known *Radula* species, we formally describe it as a new species.

**TABLE 1:** Morphological comparisons among fossil species of *Radula*.

Species	Stem	Lobe		Lobule shape	Lobule to lobe length radio	Reduced axes type	Keel to lobe length ratio	Lobe cell		Age	Source	References
		Lobe shape	Apex					Cell shape	Cell wall			
*R*. *kachinensis* sp. nov.	Slightly zig‐zagged	Elliptic to ovate, reniform	Round	elliptic, oblong	1/2	Amentulose branches	< 1/4	Quadrate to rectangular (Marginal); slightly elongate or hexagonal (Medial)	Conspicuous thickened with angular trigones	Cretaceous	Kachin amber	This paper
*R. cretacea* Bechteler, M.A.M. Renner, Schäf.‐Verw. and Heinrichs	Straight to slightly zig‐zagged	Ovate	Acute	Quadrate to trapeziform	1/5	Absent	1/4–1/3	Quadrate to rectangular, isodiametric to slightly elongate, irregularly sized (Marginal)	Unthickened with small concave trigones	Cretaceous	Kachin amber	Bechteler et al. (2017)
*R. heinrichsii* K. Feldberg, Schäf.‐Verw., M.A.M. Renner, von Konrat and A.R. Schmidt	Slightly	oval, ovate to obovate	Round	Orbicular to obovate	1/3–2/5	Amentulose branches	1/4–1/3	Quadrate to rectangular (Marginal); (Sub)isodiametric (Medial)	Slightly thickened	Cretaceous	Kachin amber	Feldberg et al. (2021); Wang et al. (2022)
*R. patrickmuelleri* K. Feldberg, Schäf.‐Verw. and M.A.M. Renner	Zig‐zagged	Oblong‐elliptic, entire to slightly crenulat	Broadly round to obtuse	Small rounded rectangular to ovate with subacute to rounded apex	< 1/5	Absent	1/6	Quadrate to rectangular; Hexagonal (Marginal); mostly isodiametric to occasionally weakly elongated (Medial)	Thin with small, triangular trigones	Cretaceous	Kachin amber	Feldberg et al. (2022)
*R. tanaiensis* K. Feldberg, Schäf.‐Verw. and M.A.M. Renner	Straight to slightly zig‐zagged	Oval to ovate	Round	Ovate to rounded trapezoid	7/10–9/10	Absent	1/2–3/5	Quadrate to rectangular (Marginal); (sub)isodiametric to slightly elongated (Medial)	Marginal cells distinctly thickened; moderately thickened, with small triangular to subnodulose trigones	Cretaceous	Kachin amber	Feldberg et al. (2022)
*R. baltica* Heinrichs, Schäf.‐Verw. and M.A.M. Renner	Straight	Rotund to broadly elliptic	Round	Quadratic to rhombiform	1/3	Microphyllous branches	Length not mentioned	Elliptic (Medial)	Medial cell wall thickened, with bulging, elongate trigones	Eocene	Baltic amber	Heinrichs et al. (2016)
*R. sphaerocarpoides* Grolle	Zig‐zagged	Elliptical to elliptical‐oblong	Round	Ovate	1/2	Absent	Length not mentioned	Isodiametric to elongated (Medial)	Cell walls thin, with triangular to subnodulose trigones	Eocene	Baltic amber	Heinrichs et al. (2016)
*R. oblongifolia* Caspary	Straight	Oblong to broadly elliptical	Round	Quadratic to rectangular	1/2–3/5	Absent	Length not mentioned	Quadrate to rectangular (Marginal); rectangular (Medial)	Cell wall thickened	Eocene or Oligocene	Baltic amber and Bitterfeld amber	Heinrichs et al. (2016)
*R. intecta* M.A.M. Renner, Schäf.‐Verw. and Heinrichs	Straight	Broadly elliptic; some lobe margins with ciliate outgrowths	Round	Subquadrate	1/5	Absent	Length not mentioned	Quadrate to rectangular (Marginal); isodiametric to slightly elongate (Marginal)	Some exterior cell walls triangular with trigones small	Miocene	Dominican amber	Kaasalainen et al. (2016)
*R. steerei* Grolle	Straight	Obliquely ovate to obliquely triangular‐ovate	Round	Shortly rectangular	1/2	Microphyllous branches	1/2	Rectangular (Marginal); isodiametric to slightly elongate (Medial)	Cells with colorless thin walls without trigones	Miocene	Dominican amber	Grolle (1987)

In *Radula*, amentulose‐like branches present primarily in subg. *Amentuloradula* and, in *R*. *prolifera* Arnell of subg. *Radula* (Yamada 1979; Devos, Renner, Gradstein, Shaw, and Vanderpoorten 2011; Renner et al. 2022; Promma et al. 2025). *Radula prolifera* is easily distinguished from *R*. *kachinensis* by its subquadrate lobules with sinuate keels and loosely imbricate to slightly distant leaf lobes (Yamada 1979). Moreover, the morphocladistic analysis of *R*. *heinrichsii*, which serves as a relevant comparative reference and bears a close resemblance to *R*. *kachinensis*, supports its placement within subg. *Amentuloradula*. The observed morphological variation within *Amentuloradula* potentially reflects early stages of evolutionary divergence (Patiño et al. 2017; Feldberg, Schaefer‐Verwimp, et al. 2021). Therefore, based on a comprehensive assessment of both morphological features and relatively phylogenetic studies, we propose that *R*. *kachinensis* should be assigned to subg. *Amentuloradula*. We note, however, some uncertainties remain regarding the fossil specimen. For instance, significant differences in stature between the primary and secondary vegetative shoots are evident in the fossil. On the primary shoot, each amentulose axis bears one or two additional amentulose branches, while those on the secondary shoot are unbranched or bear only one additional amentulose branch (Figure 4A–F). This is not a common feature of the modern species of *Amentuloradula*, whose vegetative shoots typically exhibit monomorphism regardless of branching order (pers. comm. Dr. Matthew A.M. Renner). In addition, although amentulose shoots bear one or two lateral branches at the base, they do not branch near the apex of the parent shoot, possibly because the amentulose shoot has not yet branched (pers. comm. Dr. Matthew A.M. Renner).

Summarily, the new fossil species *Radula kachinensis* sp. nov. provides valuable insights into the diversity of epiphytic liverworts during the Cretaceous period and presents a rare shoot system exhibiting three orders of branching within *Radula*. Although the fossil species described lacks detailed information on sexual reproductive features, the evidence we have provided supports its recognition as a distinct new fossil species. Additional well‐preserved specimens will further refine our understanding of *Radula*'s morphological diversity and contribute to a more detailed reconstruction of its evolutionary history.

## Author Contributions

**Xiangbo Song:** conceptualization (equal), investigation (equal), visualization (lead), writing – original draft (lead), writing – review and editing (lead). **Wen Ye:** conceptualization (equal), investigation (lead), supervision (equal), writing – original draft (equal), writing – review and editing (equal). **Zixi Wang:** conceptualization (lead), funding acquisition (lead), investigation (equal), supervision (lead), visualization (equal), writing – original draft (equal), writing – review and editing (equal).

## Conflicts of Interest

The authors declare no conflicts of interest.

## Data Availability

Data sharing not applicable to this article as no datasets were generated or analyzed during the current study.
